# Handgrip Strength as a Darwinian Fitness Indicator in Men

**DOI:** 10.3389/fpsyg.2018.00439

**Published:** 2018-04-06

**Authors:** Andrew C. Gallup, Bernhard Fink

**Affiliations:** ^1^Department of Social and Behavioral Sciences, SUNY Polytechnic Institute, Utica, NY, United States; ^2^Institute of Psychology, Georg-August University of Goettingen, Göttingen, Germany

**Keywords:** handgrip strength, sexual behavior, body morphology, attractiveness, aggression

## Abstract

Handgrip strength (HGS) is a robust measure of overall muscular strength and function, and has long been predictive of a multitude of health factors and physical outcomes for both men and women. The fact that HGS represents such a ubiquitous measure of health and vitality may reflect the significance of this trait during human evolution. This trait is also highly sexually dimorphic due to influences of androgenic hormones and fat-free body mass, suggesting that it has been further elaborated through sexual selection. Consistent with this view, research within evolutionary psychology and related fields has documented distinct relationships between HGS and measures of social and sexual behavior, especially in men. Here, we review studies across different societies and cultural contexts showing that male HGS predicts measures of aggression and social dominance, perceived formidability, male-typical body morphology and movement, courtship display, physical attractiveness, and sexual behavior and reproductive fitness. These findings underscore the value of including HGS as an independent measure within studies examining human sexual selection, and corroborate existing research suggesting that specific features of physical strength have and continue to be under positive directional selection in men.

## Introduction

Handgrip strength (HGS) is an easily obtainable and robust measure of overall muscular strength in humans (Wind et al., 2010), with highest scores typically occurring between the ages of 24 and 39 years (Mathiowetz et al., 1985). Acquired through the use of a hand dynamometer, HGS has been used to evaluate sports performance in athletes (Cronin et al., 2017) and is commonly measured within medical and rehabilitation settings to assess physical status and post-operative recovery (Innes, 1999). Low HGS is predictive of premature mortality, increased disability, and greater risk of health complications and lengthier stay following hospitalization or surgery (Bohannon, 2008). Although typically assessed in the later stages of life, HGS is even a significant predictor of mortality when measured at younger ages (Rantanen et al., 1999; Cooper et al., 2010). Among women, HGS is commonly used to assess frailty and changes in bone mineral density following menopause (Iolascon et al., 2017). In addition to well-documented links to physical health, low HGS has also been shown to predict cognitive decline within geriatric populations (Taekema et al., 2010). Thus, HGS seems to be a powerful indicator of health and vitality for both men and women, as it relates to overall physical functioning and morbidity (Sayer et al., 2006).

Although HGS appears to be equally predictive of positive health and physical outcomes in both sexes, this measure is highly sexually dimorphic with men consistently showing greater HGS than women (Mathiowetz et al., 1985; Kamarul et al., 2006). The sexual dimorphism in physical strength between men and women far exceeds the discrepancies between the sexes in terms of stature and overall body mass (Isen et al., 2014), and thus likely reflects the disproportionately higher levels of androgenic hormones (Page et al., 2005) and upper-body muscularity of men compared to women (Kallman et al., 1990; Lassek and Gaulin, 2009). While variability in HGS can be influenced by diet and specific hand exercise within clinical populations (Norman et al., 2011; Cima et al., 2013), and exposure to androgens during intrauterine development (Fink et al., 2006; but see Gallup et al., 2007), studies consistently show that HGS is strongly influenced by genetic factors (Reed et al., 1991; Fredericksen et al., 2002; Isen et al., 2014). In fact, general exercise intervention programs that improve strength in other areas tend to have little to no effect on HGS at least among frail older people (Tieland et al., 2015). Interestingly, the heritability estimates for HGS are also sexually dimorphic, ranging between 50 and 65% for adult male twins (Reed et al., 1991; Fredericksen et al., 2002; Silventoinen et al., 2008) and being considerably lower for women (30%) (Arden and Spector, 1997). Supporting a role of androgenic influences in the development of physical strength, a recent longitudinal study by Isen et al. (2014) showed that additive genetic effects accounted for far more of the variance in the development of HGS during the period of adolescence for boys (80%) than girls (28%). Consistent with this view, greater age-related declines in HGS are also found earlier in men compared to women (Vianna et al., 2007).

### Handgrip Strength as a Fitness Indicator in Men

The marked sexual dimorphism in overall HGS, combined with the distinct genetic and developmental factors influencing men and women, suggests that during human evolutionary history specific features of upper-body muscularity were further elaborated among males through sexual selection. Increased physical strength would have undoubtedly been favored within contexts of direct male–male competition and fighting (Sell et al., 2009), protection from predators (Sell et al., 2012), hunting (Apicella, 2014), and tool use and manufacture (Young, 2003). Arguably, HGS in particular, rather than other features of upper-body muscularity, would have had tremendous importance within these contexts. In regards to fighting, HGS alone is a robust predictor of ability and outcomes. For example, the correlation between HGS and ranking among amateur middleweight boxers is 0.87 (Guidetti et al., 2002). In addition, forearm strength is particularly important for traditional forms of hunting (Smith et al., 2017). Furthermore, tool use and manufacture has likely played a direct role in shaping both precision and powerful gripping during human evolutionary history (Young, 2003). Due to the vital importance of this trait within the ancestral environment, cues of upper-body muscularity and formidability seem to be important features of female mate choice among modern humans as they account for ∼70% of the variance in male bodily attractiveness (Sell et al., 2017). Thus, HGS has likely been under directional selection in men as it relates to reproductive competition.

Consistent with the sex-specific role of HGS within contexts of social and sexual competition, Gallup et al. (2007) found that HGS predicted self-reported levels of aggression, male-typical body morphology, and sexual behavior in men, while none of the variables examined were correlated with HGS in a comparable sample of women. Here, we review the latest literature to investigate the extent to which these initial findings have been replicated and extended. We focused on peer-reviewed articles in evolutionary psychology and related fields that explicitly examined relationships between HGS and measures of inter- and intra-sexual selection. Although a growing number of studies have included HGS within composite measures of upper-body strength (e.g., Sell et al., 2009; Lukaszewski and Roney, 2011; Smith et al., 2017), many fail to report on the specific connection of HGS to the dependent measures. However, in cases where HGS is parceled out of these composite measures we do report the documented effects. **Table 1** presents the findings over the last decade specifically linking HGS to measures of intra- and inter-sexual selection and reproductive fitness.

**Table 1:** Studies specifically linking HGS to measures of inter- and intra-sexual selection in men.

Variable	Source	Measure	Sample (M/F)	Country (culture)	Correlation/effect (M/F)
**Aggression and social dominance**					
*Self-reported aggression*	Gallup et al., 2007+ Archer and Thanzami, 2007 Archer and Thanzami, 2009 Gallup et al., 2010+ Shetty et al., 2016 Zhang and Reid, 2017	Maximum Maximum Averaged Maximum Maximum Averaged	82 M; 61 F 88 M 85 M 65 M; 52 F 68 M; 69 F 142 M	United States India India United States India United States	Positive (M) Positive Positive No effect Positive (M) Positive
*Self-reported victimization*	Gallup et al., 2007+ Gallup et al., 2010+	Maximum Maximum	82 M; 61 F 65 M; 52 F	United States United States	No effect Negative (M)
*Self-reported popularity*	Gallup et al., 2010+	Maximum	65 M; 52 F	United States	Positive (M)
*Aggression from face*	Gallup et al., 2010	Maximum	69 M; 93 F	United States	Positive (M; M+F rated)
*Dominance from face*	Fink et al., 2007 Windhager et al., 2011 Gallup et al., 2010	Averaged Averaged Maximum	32 M 26 M 69 M; 93 F	Germany Germany United States	Positive (F rated) Positive (F rated) Positive (M; M+F rated)
*Dominance of gait*	Fink et al., 2016a	Averaged	80 M	Germany	Strong > Weak (M+F rated)
*Self-perceived fighting ability*	Muñoz-Reyes et al., 2012 Muñoz-Reyes et al., 2015	Maximum Maximum	142 M; 146 F 152 M	Spain Chile	Positive (M+F) Positive
**Body morphology**					
*Facial masculinity*	Fink et al., 2007 Windhager et al., 2011 Van Dongen, 2014	Averaged Averaged Averaged	32 M 26 M 92 M; 112 F	Germany Germany Belgium	Positive (F rated) Positive (F rated) Positive (F; FLA)
*Male-typical body morphology*	Gallup et al., 2007 Shoup and Gallup, 2008 Sim, 2013	Maximum Maximum Maximum	82 M 38 M 94 M; 143 F	United States United States United States	Positive (SHR) Positive (SHR) Positive (SHR)
*Fluctuating asymmetry*	Sim, 2013 Fink et al., 2014 Van Dongen, 2014	Maximum Averaged Averaged	69 M; 93 F 69 M 92 M; 112 F	United States United Kingdom Belgium	Negative (F) Negative No effect
**Physical attractiveness**					
*Facial attractiveness*	Fink et al., 2007 Shoup and Gallup, 2008 Gallup et al., 2010 Van Dongen, 2014	Averaged Maximum Maximum Averaged	32 M 38 M 69 M; 93 F 92 M; 112 F	Germany United States United States Belgium	Positive (F rated) Positive (F rated) Positive (M; M+F rated) No effect (M+F rated)
*Self-perceived bodily attractiveness*	Sneade and Furnham, 2016	Maximum	145 M	United Kingdom	Positive
*Attractiveness of gait*	Fink et al., 2016a Fink et al., 2017	Averaged Averaged	80 M 80 M	Germany Germany, Chile, and Russia	Strong > Weak (F rated) Strong > Weak (F rated)
**Courtship display**					
*Dance quality and attractiveness*	Hugill et al., 2009 McCarty et al., 2013 Weege et al., 2015	Averaged Averaged Averaged	40 M 30 M 75 M; 84 F	Germany United Kingdom Germany	Positive (F rated) Positive (M+F rated) Positive (M; M+F rated)
**Reproductive fitness**					
*Age of sexual intercourse*	Gallup et al., 2007 Shoup and Gallup, 2008 Varella et al., 2014 Sneade and Furnham, 2016	Maximum Maximum Averaged Maximum	82 M; 61 F 38 M 91 M; 94 F 145 M	United States United States Brazil and Czechia United Kingdom	Negative (M) No effect Negative (M) Negative
*Total sex partners and promiscuity*	Gallup et al., 2007 Shoup and Gallup, 2008 Varella et al., 2014 Sneade and Furnham, 2016	Maximum Maximum Averaged Maximum	82 M; 61 F 38 M 91 M; 94 F 145 M	United States United States Brazil and Czechia United Kingdom	Positive (M) Positive Positive (M); Negative (F) Positive
*Self-reported mate value*	Archer and Thanzami, 2009 Muñoz-Reyes et al., 2015	Averaged Maximum	85 M 152 M	India Chile	Positive Positive
*Number of children (self-report)*	Atkinson et al., 2012 Apicella (personal communication)	Maximum Averaged	36 M; 54 F 52 M; 66 F	Namibia (Himba) Tanzania (Hadza)	Positive (F) Positive (M)
**Other fitness-relevant measures**					
*Hunting reputation*	Apicella, 2014	Averaged	52 M	Tanzania (Hadza)	Positive (F rated)

*^+^Studies evaluated both middle and high school behavior, but high school findings are shown here. FLA, facial landmark analysis.*

### Measures of Intrasexual Selection

A large number of studies have examined the connection between HGS and measures of aggression and social dominance. Specifically, self-reported aggression during later adolescence and young adulthood has been found to be positively correlated with HGS in men but not women (Archer and Thanzami, 2007, 2009; Gallup et al., 2007; Shetty et al., 2016; Zhang and Reid, 2017; but see Gallup et al., 2010). Other studies reported positive correlations between HGS and perceived aggression and social dominance based on independent ratings of male faces (Fink et al., 2007; Gallup et al., 2010; Windhager et al., 2011). In a study investigating the relationship between HGS and male walking movements, individuals with high HGS were perceived as more dominant than weaker men (Fink et al., 2016a). Only two studies have investigated the connection between HGS and victimization (i.e., being the target of aggression from peers), showing mixed results for males and no effect for females (Gallup et al., 2007, 2010). One study examining popularity showed a positive correlation among high-school aged boys but no connection for girls, while the opposite was true during middle school (Gallup et al., 2010). Combined these findings suggest that HGS is a good indicator of social dominance, but only among older adolescent and adult men. Consistent with this view, it has been speculated that the importance of physical strength should increase within male–male competitive social contexts where reproductive activities are more salient and disparities in physical size and stature are less pronounced following advanced pubertal development (see Gallup et al., 2010).

However, cross-sectional studies investigating the correlation between HGS and aggression have limitations in disentangling developmental causality. In a longitudinal study, Isen et al. (2015) demonstrated that male antisocial tendencies temporarily precede their physical formidability. Boys (but not girls) with greater aggressive-antisocial tendencies in childhood were found to attain larger increases in HGS later in adolescence. Thus, for males, individual differences in aggression seem to be linked to the development of HGS. In accord with sexual selection theory, the authors concluded that antisocial-aggressive dispositions in childhood may prepare males for intrasexual competition in young adulthood.

Two studies have assessed the relationship between HGS and self-perceived fighting ability, one with both sexes during middle and later adolescence and one with just late adolescent men, and in all cases, there was a positive correlation (Muñoz-Reyes et al., 2012, 2015). Previous research has already demonstrated that males with greater HGS are better fighters (Guidetti et al., 2002), and a recent study showed HGS specifically increases among men following exposure to challenges (i.e., viewing aggressive rugby videos) (Ribeiro et al., 2016). Thus, HGS likely plays a specific functional role in direct male–male competition.

### Body Morphology

Studies that have examined the connection between HGS and male-typical features of facial and body morphology have predominantly included just male participants. Fink et al. (2007) first reported positive correlations between HGS and female ratings of male facial masculinity and dominance after controlling for the effects of age and body weight. Although this study documented high inter-correlations between the attributes (*r* > 0.60), it did not examine connections between HGS and distinct facial characteristics. Employing a geometric morphometrics approach, Windhager et al. (2011) showed that the faces of men with greater HGS tended to have wider eyebrows and a prominent jawline (see also, Holzleitner and Perrett, 2016). Moreover, shape regressions revealed that facial shape of males with high HGS showed strong relationships with female perceptions of masculinity and dominance. However, when using facial landmarks to determine masculinity scores (instead of human ratings), Van Dongen (2014) reported a positive correlation with HGS in women but not men. Taken together, studies that provide a more complete approach to the assessment of facial morphology, i.e., considering facial shape as a single geometric whole rather than relying on measures of specific angles or ratios, suggest morphological differences within the faces of physically strong men. In addition, the relationships seem to be more robust for female assessments of male faces.

When examining the relationship between HGS and body configuration, study results consistently show a positive association with male-typical features. Three studies have used shoulder-to-hip ratio (SHR) as a principle measure of male body morphology. Higher SHRs produce a more wedge-shaped torso, which is correlated with testosterone (Kasperk et al., 1997) and found to be attractive by women (Dijkstra and Buunk, 2001). Thus, SHR represents an informative anthropomorphic measurement of male upper-body configuration. As predicted, HGS has been shown to be positively correlated with SHR in all samples, including one with female participants (Gallup et al., 2007, 2010; Sim, 2013).

Studies examining the connection between HGS and fluctuating asymmetry (FA) – a measure of developmental stability and health – provide much more inconsistent results. The first study to investigate this relationship measured six bilateral traits, and found that HGS was negatively correlated with FA in women but not men (Sim, 2013). Combining measurements across 12 paired traits, HGS was found to be negatively correlated with FA within a subsequent sample of men (Fink et al., 2014). The only other study specifically examined facial FA, and found that HGS was not a predictor of this variable in either men or women (Van Dongen, 2014). Thus, while HGS is linked to male-typical morphology, future research is needed in the area of FA.

### Physical Attractiveness and Courtship Display

Studies assessing the relationship between HGS and facial attractiveness among men have consistently revealed significant positive correlations when rated by women (Fink et al., 2007; Shoup and Gallup, 2008). Gallup et al. (2010) investigated this connection using mixed-sex ratings of senior high school yearbook photos, finding a marginally significant correlation between HGS and attractiveness for men but not women. Similarly, when combining health and attractiveness ratings together, which were highly correlated, there was a significant positive correlation with HGS for men only. However, the relationship between male HGS and facial attractiveness has not held up across all studies. In one study with mixed-sex raters, HGS failed to predict facial attractiveness in either men or women (Van Dongen, 2014).

Studies with only male participants have consistently linked HGS to measures of bodily attractiveness. Recently, HGS was shown to be positively correlated with self-reported ratings of overall physical attractiveness (Sneade and Furnham, 2016). In two other recent studies, men with high HGS were perceived to have more attractive gaits compared with weaker men, as rated by female samples from a set of diverse cultural backgrounds (Fink et al., 2016a, 2017).

Three studies have also specifically investigated the connection between measures of HGS and dancing quality and attractiveness (Hugill et al., 2009; McCarty et al., 2013; Weege et al., 2015). Dance and other types of bodily movement seem to represent an important aspect of courtship display across different cultures (Kaeppler, 1978; Fink et al., 2015). Hugill et al. (2009) found that female ratings of both dance attractiveness and assertiveness among men were significantly correlated with their HGS. McCarty et al. (2013) performed biomechanical analyses on a separate sample of male dancers, finding that both male and female ratings of men’s dance quality were significantly predicted by HGS. In particular, stronger men that displayed larger, more variable and faster arm movements were rated as better dancers. In another study, this time including both male and female dancers for comparison, it was shown that opposite-sex ratings of dance attractiveness were correlated in men but not women (Weege et al., 2015). In total, HGS appears to be a good predictor of physical attractiveness among men.

### Sexual Behavior and Reproductive Fitness

While the studies referenced above support a link between male HGS and reproductive competition, measures of sexual behavior and offspring production obviously represent more direct indicators of fitness. To date, four studies have investigated the association between HGS and self-reported sexual behavior (two including both sexes: Gallup et al., 2007; Varella et al., 2014; and two with just men: Shoup and Gallup, 2008; Sneade and Furnham, 2016). In all cases, spanning a variety of industrialized cultures, HGS in men was positively correlated with lifetime number of sex partners or specific measures of promiscuity. In studies including women, HGS either showed no relationship (Gallup et al., 2007) or was actually negatively correlated with these measures (Varella et al., 2014). Male HGS was also shown to predict an earlier onset of male sexual behavior within all but one of these studies (see Shoup and Gallup, 2008). In two additional studies on men, HGS was positively correlated with self-assessments of mate value (Archer and Thanzami, 2009; Muñoz-Reyes et al., 2015).

In the only study to specifically report the connection between HGS and self-reported offspring production (Atkinson et al., 2012), a pattern opposite to that of the aforementioned findings for sexual behavior was observed: among the Himba, a group of semi-nomadic, pastoralists of Namibia, HGS was shown to be positively correlated with the number of living children in women, while no relationship was observed for men. This relationship for women was particularly true among older individuals, which was interpreted under the lens of the Grandmother Hypothesis (e.g., Hawkes et al., 1998). While this may represent an interesting culture-specific effect within this traditional group, the authors highlight the need for paternity data to confirm this null result for men given the particularly high rates of extra-pair paternity within this population (Scelza, 2011).

In a study of the Hadza hunter-gatherers of Tanzania, Apicella (2014) showed that a composite measure of male upper-body strength significantly predicted both hunting reputation (as measured by resident women) and self-reported reproductive success in terms of offspring production. When HGS was parceled out from the composite measure to specifically analyze predictors of hunting reputation, it was found that the positive relationship with upper-body strength was “driven by HGS of the right hand” (Apicella, 2014, p. 513). The specific relationship between HGS and reproductive fitness was not reported in this paper. However, in a personal communication, subsequent analyses revealed that HGS alone significantly positively predicted the number of living offspring and negatively predicted child loss among Hadza men (Apicella, personal communication). That is, men with high HGS self-reported more living children and the offspring from these stronger fathers were less likely to die. Furthermore, an unpublished dataset showed no relationship between HGS and reproductive success among Hadza women (Apicella, personal communication). Similar to the Himba population referenced above, however, these findings for men should be interpreted with caution until supported by paternity data.

## Concluding Remarks and Future Research

The connections between HGS and overall health and vitality among both men and women have long been recognized, but only over the past decade have studies begun to identify the sex-specific relationships between HGS and measures of intra- and inter-sexual selection. Although variability is present within this literature, and there is a disproportionate representation of male to female participants across studies, a fairly consistent pattern emerges: HGS correlates with numerous measures involved in social and sexual competition in men, and typically fails to correlate with these measures among women. The studies reported here span a variety of measurement techniques, include samples from a wide representation of cultures and geographic locations, and many of the specific findings have been replicated in independent laboratories. We propose that the predominantly male-specific nature of these effects, combined with the sexually dimorphic developmental and genetic factors contributing to HGS, stem from ancestral conditions in which this trait was more directly linked to survival and reproduction among men, particularly within contexts of fighting, hunting, protection and provisioning of kin, and tool use and manufacture.

As initially pointed out by Gallup et al. (2007), the sex asymmetry for HGS correlating with measures of social and sexual competition, but not health status, may be a result of a primitive division of labor that emerged within hunter-gatherer societies placing a premium on the maintenance and further elaboration of male HGS in competition for securing resources. Within the only study to date that specifically assessed the connection between HGS and reproductive fitness among hunter-gatherers (Apicella, 2014), this trait was the single best predictor of female rated hunting reputation and was positively correlated with offspring production and survival among Hadza men (Apicella, 2014, personal communication). These results are consistent with findings from contemporary samples from the United States, Europe, and South America, in which HGS is a reliable indicator of self-reported sexual behavior and mate value among men. However, the inverse effects observed among the pastoralist Himba of Namibia make it clear that further research (using paternity data) is necessary to elucidate the connection between HGS and reproductive fitness across various cultures. Future research could also examine the relationship between HGS and measures of genetic quality, including the previously hypothesized connection between HGS and semen quality (Gallup and Gallup, 2016). Given the growing number of studies linking male HGS to specific measures of personality and psychological well-being (Fink et al., 2010, 2016b; Hugill et al., 2011; Sneade and Furnham, 2016), another potentially fruitful area of research would be to examine how HGS correlates with status seeking, ambitious/industriousness, and competitiveness, as well as measures of resource acquisition among men and women within developed countries (i.e., income, employment status, and ranking/promotion).

To date, HGS has proven to be a valuable measure within the evolutionary behavioral sciences and many areas have yet to be fully explored. Although there is a trend for studies to include HGS within composite measures of upper-body strength, for reasons outlined above we suspect HGS alone might be the most important measurement of male strength. Thus, we encourage researchers to assess the specific effects of HGS within future studies and work toward developing a standardized technique for assessing this preeminent trait.

## Author Contributions

All authors listed have made a substantial, direct and intellectual contribution to the work, and approved it for publication.

## Conflict of Interest Statement

The authors declare that the research was conducted in the absence of any commercial or financial relationships that could be construed as a potential conflict of interest.
